# Supplementary material for: Comparison of acute appendicitis before and within lockdown period in COVID-19 era: A retrospective study from rural Nepal
Source: PLoS One. 2021 Jan 6;16(1):e0245137. doi: 10.1371/journal.pone.0245137 (PMC7787439; doi:10.1371/journal.pone.0245137)
Supplement: Attachment — Submitted filename: Response to Reviewers.docx [file pone.0245137.s002.docx]

11/23/2020

The Editor in Chief

PLOS One

**Re: Resubmission of the manuscript along with rebuttal letter as per suggestions**

Sir

Please kindly find the below mentioned response for the suggestions as provided by the esteemed editors and reviewers. I have tried my best to incorporate the shortcomings as suggested and meet upon the expectations. Please feel free to contact me for any corrections needed.

Sincerely

Suman Baral

(P.S: The responses go as below)

**Editorial comments**

1.Please ensure that your manuscript meets PLOS ONE's style requirements, including those for file naming.

\# Response: Ensured: Title page and manuscript page changed as suggested

2. Please provide additional details regarding participant consent. In the ethics statement in the Methods and online submission information, please ensure that you have specified (1) whether consent was informed and (2) what type you obtained (for instance, written or verbal, and if verbal, how it was documented and witnessed). If your study included minors, state whether you obtained consent from parents or guardians. If the need for consent was waived by the ethics committee, please include this information.

If you are reporting a retrospective study of medical records or archived samples, please ensure that you have discussed whether all data were fully anonymized before you accessed them and/or whether the IRB or ethics committee waived the requirement for informed consent. If patients provided informed written consent to have data from their medical records used in research, please include this information.

 # Response: As the nature of the study was record based and retrospective, consent was waived by the ethical committee of the institute and was not obtained and data too were anonymized (Sentence added: 8-10^th^ line- Methods)

3. Please include the date(s) on which you accessed the databases or records to obtain the data used in your study.

\# Response: from July 1 to July 4 2020 (Phrase added on Line 7- Methods)

4. We noted in your submission details that a portion of your manuscript may have been presented or published elsewhere.

"The manuscript has been uploaded as pre-print in Research Square."

Please clarify whether this publication was peer-reviewed and formally published. If this work was previously peer-reviewed and published, in the cover letter please provide the reason that this work does not constitute dual publication and should be included in the current manuscript.

##### # Response: The current manuscript has been uploaded as a pre-print in the Research Square and the assigned DOI number is [10.21203/rs.3.rs-47510/v2](https://doi.org/10.21203/rs.3.rs-47510/v2). However, no peer review has been done and this does not constitute the dual publication. (added in Acknowledgement)

##### Reviewers Comments

##### # Reviewer 1

##### 1. Table nr. 1 if possible, should be redone, and the number of rows decreased as only the essential variables should be compared. The others could be added in plain text.

##### Response# Modified as suggested!

##### # Reviewer 2

##### 1. The authors attempt to elucidate the topic of "Comparison of acute appendicitis before and within COVID-19 era", but not all of the patients had been tested for coronavirus in such a small sample size. In this situation, it should be hard to make any conclusion on this point as your title.

##### Response# Title modification done:

**Old title**: Comparison of acute appendicitis before and within COVID-19 era: A retrospective study from rural Nepal

**New title**: Comparison of acute appendicitis before and within lockdown period in COVID-19 era: A retrospective study from rural Nepal

\# Reviewer 3

1. In Group A, more patient received open appendectomy rather than laparoscopic appendectomy (75 VS 9). In Group B, no patient underwent laparoscopic operation (47 VS 0). Since the mainstream of appendectomy around the world is through endoscope due to lower/ similar complication rate and shorter hospital stay, why did few patients receive laparoscopic surgery, particularly in Group B? Notably, it’s seems to be more controllable for infection during endoscopic surgery rather than open surgery. This topic shall be addressed.

\# Comment: With the advent of first minimal invasive surgery as laparoscopic appendectomy in 1983 by Semm, the treatment of this most common surgical emergencies has seen an immense shift of treatment procedure from open approach to laparoscopy, however open approaches have not been abandoned in low middle-income countries like Nepal. Instead, we could see a surge of open surgeries in comparison to laparoscopy especially in the rural parts of the country. Potential advantages of early return to work, minimal hospital stay, minimized post-operative surgical site infections have been documented in literatures as the beneficial outcomes in comparison to open appendectomies, however the cost factor plays a critical role in low economies where surgical health is still primitive and laparoscopic advancements in rural part of the country is still to perish. Also, the surgical choice is to be decided by the patient and still the traditional open approaches are considered by them. Lower number of laparoscopies before COVID-19 and no any laparoscopic appendectomy during lockdown was evident in our clinical practice. The main reason behind this low digit was due to patient reluctance for laparoscopic surgery and cost factors which surpasses open appendectomy. However, during lockdown, laparoscopy was completely abandoned at our setting as various controversies existed regarding aerosol generating procedures and safety of the heath care workers too were inconclusive.: *Line 30- 44 added in Discussion*

2. According to comment 1, the duration of surgery, post OP hospital stay, or perforation rate shall be discussed in open surgery or laparoscopic operation in Group A and Group B, respectively. That is, in addition to infection control or additional preparation, did the mean time duration of surgery in Group B longer than the time in Group A may result from the type of operation? The author shall clarify this issue.

\# Response: Various literatures have shown that the duration of surgery is longer in laparoscopy group than open techniques, however our experience suggests no any significance regarding the technique of the surgery as both the laparoscopic and open appendectomies took almost similar time duration. Considering the teaching institute, most of the open appendectomies were performed by the surgical residents under supervision while minimal laparoscopies performed were done by the experienced consultants which also may be one of the factors of increasing time duration of surgery in group B where no laparoscopy was considered. *Line 50-56 added in discussion*

3. In Table 2 describing the relationship between the presence of fecalith and perforation, why the group A is only 37 people and group B is 47 people? The case number is different from cases included? What is the missing cases?

\# Response: Missing cases (N= 8) were managed conservatively. They were not operated.

4. The author concluded the number of cases of acute appendicitis increase after pandemic of COVID-19. However, the author did not offered a substantial evidence of the growing number of cases. Similarly, the author concluded that the ratio of complicated case (perforation) increase after pandemic of COVID-19. However, according to the Table 1, there is no statistically significant difference between the differences of perforation rate in two group.

\# Response:

1. In contrary, we could see a greater number of patients surfacing to the emergencies and getting operated in comparison to the same time frame before this contagious disaster. The valid reason for this disparity, though small in number, could be due to closure of private hospitals around the area after the lockdown was initiated, from where the patient turns up and the pooling occurred at our institute as this serves as one of the tertiary centers in the region. (Line 4-9, Discussion)
2. The lesser time duration and lower number of cases are the limitations of the present study. COVID- 19 is a new disease and we could not find similar studies from the region to compare and discuss the findings. We believe as the duration of the study is 3 months pre and post COVID era within lockdown and with the limited number of cases, the statistics may not be fully relied upon as this may be misleading as exemplified in the perforation rates between two groups. Also, this is a single center analysis of the patients with a smaller sample size which might not cover all the demographic and clinical aspects of the cohorts. Line 83-90, Discussion)
