# Supplementary material for: Comparison of acute appendicitis before and within lockdown period in COVID-19 era: A retrospective study from rural Nepal
Source: PLoS One. 2021 Jan 6;16(1):e0245137. doi: 10.1371/journal.pone.0245137 (PMC7787439; doi:10.1371/journal.pone.0245137)
Supplement: Attachment — Submitted filename: Response to Reviewers1.docx [file pone.0245137.s003.docx]

19/12/2020

The Editor in Chief

PLOS One

**Re: Resubmission of the manuscript along with rebuttal letter as per suggestions**

Sir

Please kindly find the below mentioned response for the suggestions as provided by the esteemed editors and reviewers. I have tried my best to incorporate the shortcomings as suggested and meet upon the expectations. Please feel free to contact me for any corrections needed.

Sincerely

Suman Baral

(P.S: The responses go as below)

**Reviewer 1 Comments**

1. In the results:
   The authors mention: Leukocytosis more than 10000/mm3 was seen in 20 patients in group A while 39 patients showed increased WBC (White
   Blood Cells) count in group B which was statistically significant.
   The authors need to be more careful with words: leukocytosis and while blood cells are two different things. Please change accordingly.

\# Response: Sentence changed

Leukocytosis more than 10000/mm^3^ was seen in 20 patients in group A while 39 patients showed leukocytosis in group B which was statistically significant. (P= 0.002)

2. There are multiple english mistakes in the text. It needs to be read by a native speaker and corrected. I have highlighted some of them in the attached document. Also the article contains very long sentences especially in the discussion segment, some of them have 4-5 rows and very hard to follow. Please change accordingly.
   Some mentions lack citations. I have highlighted them in the text.

\# Response: As suggested- Changes made.

**Reviewer 4 Comments**

1. The article does highlight the impact of Covid-19 on the management of the most common surgical emergency, that is AA. The discussion goes on and on and there are a few things that have been repeated in the intro as well as the discussion. As far as the sample size is concerned, i believe it is too small a sample to reach to a definite conclusion.

\# Response: Regarding the sample size, I too believe the number of cases is small, but considering the time duration and being a single center comparison study, the number of cases seems logical and this has been the limitation of our study.

2. The results say that there was increased pain and increased number of cases with perforation in Group B. Moreover, the duration of surgery was increased in Group B. But these points are not addressed when the results say that the duration of hospital stay was decreased in Group B and that too with a high statistical difference of almost a day.

\# Response: Duration of hospital stay tend to decrease in group B patients in our study with statistical significance in comparison to group A. Delayed presentation to hospital along with complicated appendicitis like perforation seem to have prolonged hospital stay in various literatures,[17, 18] however, this was not evident in our context especially during the time of pandemic. This could be due to patients willing to get discharged early once operated if feasible, minimizing the risk of protraction of the virus from other patients who have been hospitalized. Also, this practice allowed the rapid turnover of the patients allowing void of the beds that may be required in times of crises if surge of the COVID cases were to be seen in the forthcoming days. (Line added- Discussion- Paragraph 3 – Line 17-25)

3. Is there any association of presence of fecalith with Covid-19 or it is just a mere coincidence?

\# Coincidence may be! No any studies were found in the literature regarding this matter.
